# Optimizing parameters for clinical-scale production of high IL-12 secreting dendritic cells pulsed with oxidized whole tumor cell lysate

**DOI:** 10.1186/1479-5876-9-198

**Published:** 2011-11-14

**Authors:** Cheryl L-L Chiang, Dawn A Maier, Lana E Kandalaft, Andrea L Brennan, Evripidis Lanitis, Qunrui Ye, Bruce L Levine, Brian J Czerniecki, Daniel J Powell Jr, George Coukos

**Affiliations:** 1 Ovarian Cancer Research Center, University of Pennsylvania, 421 Curie Boulevard, Philadelphia, PA 19104, USA; 2 Clinical Cell and Vaccine Production Facility, Hospital of University of Pennsylvania, 3400 Spruce Street, Philadelphia, PA 19104, USA; 3 Department of Surgery, University of Pennsylvania Medical Center, 3400 Spruce Street, Philadelphia, PA 19104, USA

## Abstract

**Background:**

Dendritic cells (DCs) are the most potent antigen-presenting cell population for activating tumor-specific T cells. Due to the wide range of methods for generating DCs, there is no common protocol or defined set of criteria to validate the immunogenicity and function of DC vaccines.

**Methods:**

Monocyte-derived DCs were generated during 4 days of culture with recombinant granulocyte-macrophage colony stimulating factor and interleukin-4, and pulsed with tumor lysate produced by hypochlorous acid oxidation of tumor cells. Different culture parameters for clinical-scale DC preparation were investigated, including: 1) culture media; 2) culture surface; 3) duration of activating DCs with lipopolysaccharide (LPS) and interferon (IFN)-gamma; 4) method of DC harvest; and 5) cryomedia and final DC product formulation.

**Results:**

DCs cultured in CellGenix DC media containing 2% human AB serum expressed higher levels of maturation markers following lysate-loading and maturation compared to culturing with serum-free CellGenix DC media or AIM-V media, or 2% AB serum supplemented AIM-V media. Nunclon™Δ surface, but not Corning^® ^tissue-culture treated surface and Corning^® ^ultra-low attachment surface, were suitable for generating an optimal DC phenotype. Recombinant trypsin resulted in reduced major histocompatibility complex (MHC) Class I and II expression on mature lysate-loaded DCs, however presentation of MHC Class I peptides by DCs was not impaired and cell viability was higher compared to cell scraping. Preservation of DCs with an infusible cryomedia containing Plasma-Lyte A, dextrose, sodium chloride injection, human serum albumin, and DMSO yielded higher cell viability compared to using human AB serum containing 10% DMSO. Finally, activating DCs for 16 hours with LPS and IFN-γ stimulated robust mixed leukocyte reactions (MLRs), and high IL-12p70 production *in vitro *that continued for 24 hours after the cryopreserved DCs were thawed and replated in fresh media.

**Conclusions:**

This study examined criteria including DC phenotype, viability, IL-12p70 production and the ability to stimulate MLR as metrics of whole oxidized tumor lysate-pulsed DC immunogenicity and functionality. Development and optimization of this unique method is now being tested in a clinical trial of autologous oxidized tumor lysate-pulsed DC in clinical-scale in recurrent ovarian, primary peritoneal or fallopian tube cancer (NCT01132014).

## Background

Dendritic cells (DCs) are under clinical investigation as a form of cancer therapy due to their unique ability to elicit tumor-specific T cell responses. Encouraging immunologic and therapeutic responses have been observed in cancer patients treated with DC vaccines, leading to an increased interest in developing protocols for clinical-scale production of DCs for the clinic. Human DCs exist in many different subsets. Monocyte-derived DCs (MoDCs) are the most commonly used DC subset in the clinic [1-3] as it is relatively easy to obtain billions of monocytes from peripheral blood mononuclear cells (PBMCs) collected by leukapheresis. Several methods are available to enrich monocytes from PBMCs, such as plastic adherence, immunomagnetic separation and elutriation. In particular, the use of a counter-current centrifugal elutriation system that can process up to 20 × 10^9 ^PBMCs within one hour, is extremely convenient for clinical-scale isolation of monocytes and subsequent generation of DCs for clinical trials [2-4]. This approach can also be easily performed in conformity with Good Manufacturing Practice (GMP) guidelines, circumvents the need for multiple blood drawings from patients to generate sufficient DCs, and saves time and expense by eliminating plastic adherence or magnetic selection steps [5-7].

In many previous clinical trials of DC vaccines, DCs were cultured for 7 days before antigen loading, maturation and administration [8-15]. Reducing the time, labor and expense for DC generation is necessary to move DC vaccines to later phase clinical trials [16-18]. To this end, we have recently demonstrated that MoDCs cultured from an elutriated leukapheresis product for 4 days (Day-4 DCs) and pulsed with UVB-irradiated or freeze-thawed tumor cell lysate, produced higher levels of IL-12p70 and IP-10 upon activation with bacterial lipopolysaccharide (LPS) and interferon (IFN)-γ compared to MoDCs cultured for 2 or 7 days [Cheryl L-L, Chiang, Andrea R. Hagemann, Rachel Leskowitz, Rosemarie Mick, Thomas Garrabrant, Brian J. Czerniecki, Lana E. Kandalaft, Daniel J. Powell Jr. and George Coukos: Day 4 myeloid-derived dendritic cells pulsed with whole tumor lysate are highly immunogenic and elicited potent anti-tumor responses, submitted]. Moreover, these mature lysate-loaded Day-4 DCs were highly immunogenic and stimulated the strongest allogeneic T cell proliferation, while inducing equally potent specific anti-ovarian tumor responses as Day-7 'classical DCs' in T cells obtained from healthy volunteers and ovarian cancer patients

Whole tumor cells are a suitable source of tumor antigens for loading DCs in order to prime both CD4^+ ^T helper and CD8^+ ^cytotoxic T cells [19]. As whole tumor lysates provide the full repertoire of characterized and uncharacterized tumor-associated antigens for both CD4^+ ^and CD8^+ ^T cells, this allows for parallel presentation of antigens to both T cell types to generate stronger primary immune responses and to prevent the emergence of tumor escape variants. In addition, the presence of CD4^+ ^T cells helps in promoting long-term CD8^+ ^T cell memory [20-22]. Previous work has investigated the use of necrotic whole tumor cells as a source of antigens for DCs. In particular, hypochlorous acid (HOCl) was used to induce tumor cell necrosis and enhance the immunogenicity of tumor cells [23,24]. MoDCs readily engulfed HOCl-oxidized SKOV3 ovarian tumor cells, and when matured with either monophosphoryl lipid A or anti-CD40 activating antibody, they could stimulate strong T cell responses directed against specific ovarian tumor-associated antigens such as HER-2/neu and MUC1. On the other hand, MoDCs loaded with tumor cells killed with non-oxidative methods such as heat or hydrochloric acid (HCl) did not prime ovarian-specific T cell responses. These results were further supported by a mouse melanoma model where mice vaccinated with bone marrow-derived DCs loaded with oxidized syngeneic B16.F10 melanoma cells developed a T cell response against melanoma and specifically against tyrosinase-related peptide 2. These tumor-specific responses were not seen in mice treated with bone marrow-derived DCs loaded with heat- or HCl-killed B16.F10 melanoma [25].

With these encouraging results, preparations were made to take Day-4 MoDCs, loaded with HOCl-oxidized autologous ovarian tumor cells, to the clinic. This study sought to optimize the various production parameters, such as the type of media and culture vessels for DC generation, method of harvesting mature tumor lysate-loaded DCs, and freezing methods of these DCs as an 'off-the-shelf' vaccine product. It also aimed to examine and define parameters for optimal DC vaccine production and potency assessment such as DC phenotype, IL-12p70 production, and mixed leukocyte reactions (MLRs). The following findings have facilitated the development of a protocol for clinical-scale production of DC-whole tumor lysate vaccines currently being evaluated in a clinical trial in ovarian, peritoneal and fallopian tube cancers.

## Materials and methods

### Generation of DCs from fresh monocytes

For research-scale experiments, DCs were prepared from fresh PBMCs obtained from 6 healthy donors who had given written informed consent to an Institutional Review Board (IRB)-approved tissue procurement study at the University of Pennsylvania Human Immunology Core Facility. Monocytes from PBMCs were isolated by negative selection using the RosetteSep^® ^Human Monocyte Enrichment Cocktail kit (STEMCELL Technologies Inc., Vancouver, Canada) and cultured at 1 × 10^6 ^cells/ml in serum-free AIM-V media (therapeutic grade; Invitrogen, Carlsbad, CA) supplemented with 2 mM L-glutamine, 100 units/ml penicillin, and 100 μg/ml streptomycin (all from Cellgro, Manassas, VA), or in AIM-V media containing 2% human AB serum (Valley Biomedical Inc., Winchester, VA). Additional media tested included serum-free CellGenix DC media (GMP grade; CellGenix Technologie Transfer GmbH, Freiburg, Germany) that was supplemented with 2 mM L-glutamine, 100 units/ml penicillin and 100 μg/ml streptomycin, or CellGenix DC media containing 2% human AB serum. Monocytes were cultured in the presence of recombinant research grade human granulocyte-macrophage colony stimulating factor (GM-CSF; 500 or 1000 IU/ml) and interleukin-4 (IL-4; 250 or 500 IU/ml), both purchased from PeproTech (Rocky Hill, NJ). For clinical-scale DC preparations, fresh elutriated monocytes were obtained from PBMCs of 2 different healthy donors in the Clinical Cell and Vaccine Production Facility (CVPF) at the Hospital of the University of Pennsylvania. Monocytes were cultured using Nunclon™Δ Surface 1-tray Cell Factories at 1 × 10^6 ^cells/ml in CellGenix DC media containing 2% human AB serum, recombinant clinical grade GM-CSF (Leukine^®^, Bayer Healthcare Pharmaceuticals, Wayne, NJ) and animal-free research grade IL-4 (R&D Systems, Inc., Minneapolis, MN).

At the end of the 4-day culture period, the surface expression of CD11c, CD14 and HLA-DR on these differentiated immature DCs were determined and the purity was found to be >98%. HOCl-oxidized SKOV3 lysate was added to the DCs at a cell ratio of 1:1 for 20 to 24 h in the presence of fresh GM-CSF (500 IU/ml, of same origin as above depending on the culture scale). On day 5, lysate-loaded DCs were activated with LPS (60 EU/ml) and IFN-γ (2000 IU/ml) for 6 to 16 h. In research-scale DC preparations, research grade LPS (*Escherichia coli; *Sigma-Aldrich Corp.) and research grade IFN-γ (PeproTech, Rocky Hill, NJ) were used. For activation of clinical-scale DC cultures, we used clinical grade IFN-γ (Intermune, San Francisco, CA) and clinical grade LPS (*Escherichia coli *O:113; U.S. Standard Reference Endotoxin) which was a kind gift from Dr. Anthony Suffredini from the Clinical Center, National Institutes of Health, Bethesda, MD.

On day 6, mature lysate-loaded DCs were harvested, and analyzed for their phenotype and IL-12p70 production. In addition, DCs were cryopreserved at 5 × 10^6 ^cells/ml for further use. Before transitioning to clinical-scale cell factory culture system, optimal DC culture conditions were determined by setting up multiple research-scale DC preparations in T25 cm^2 ^tissue culture flasks that had the same coated-surface properties as the cell factories. Nunclon™Δ Surface T25 cm^2 ^tissue culture flasks with filter caps and Nunclon™Δ Surface 1-tray Cell Factories were purchased from Thermo Fisher Scientific Inc., Rochester, NY, USA. Corning^® ^cell culture T25 cm^2 ^tissue culture flasks with vented caps and Corning^® ^cell culture ultra-low attachment T25 cm^2 ^tissue culture flasks with vented caps were obtained from Sigma-Aldrich Corp., St. Louis, MO.

### HOCl-oxidation of whole tumor cells and preparation of tumor lysate

SKOV3 ovarian carcinoma cell line is HLA-A3^+^, A2^-^, HER-2/neu^+^, MUC1^+^. It was maintained in DMEM that was supplemented with 10% heat-inactivated fetal bovine serum (FBS, Invitrogen), 2 mM L-glutamine, 100 units/ml penicillin, and 100 μg/ml streptomycin (all from Cellgro), and was routinely tested to be free of Mycoplasma. Treatment of SKOV3 tumor cells with HOCl has been previously described [23,24]. Briefly, 60 μM of HOCl solution was prepared by diluting the stock sodium hypochlorite solution (NaOCl, reagent grade, available chlorine 10-15%; Sigma-Aldrich Corp.) with DPBS (Cellgro) and added immediately to SKOV3 tumor cells at a cell density of 1 × 10^6^/ml. The tumor cell suspension was incubated for 1 h at 37°C, 5% CO_2 _with gentle agitation after every 30 min to induce oxidation-dependent tumor cell death. After that, tumor cells were harvested, washed twice with DPBS and resuspended at 1 × 10^7 ^cells/ml in appropriate DC media for 6 cycles of freeze (either with dry ice for ≥ 20 min or at -80°C for ≥ 1 h) and thaw at room temperature to complete fragmentation (determined by Trypan blue staining) before loading onto DCs.

### DC harvesting

After stimulation with LPS and IFN-γ for 6 to 16 h, some of the mature lysate-loaded DCs were harvested using either TrypLE™ Select animal-free tyrpsin replacement (Gibco, Carlsbad, CA) or with cold DPBS and cell scrapers (BD Falcon™, San Jose, CA). For harvesting with TrypLE™ Select animal-free tyrpsin replacement, cell culture media was removed and cells incubated with 1 ml of TrypLE™ Select per T25 cm^2 ^flask at 37°C, 5% CO_2 _for 5 to 10 min. Then the cells were collected by gentle tapping and washed twice with DPBS before use. For harvesting with cold DPBS and cell scraping, cell culture media was first removed and 5 ml of cold DPBS was added to each T25 cm^2 ^flask. The DC cultures were incubated at 4°C for 30 min and a cell scraper used to remove the remaining attached DCs. DCs were then washed twice with DPBS before use.

### DC phenotyping

After coculturing DCs with HOCl-oxidized SKOV3 lysate for 20 to 24 h, LPS and IFN-γ were added for 16 h before harvesting the DCs for phenotypic analysis. DCs were harvested using TrypLE™ Select animal-free trypsin replacement or cold DPBS with scraping as described above. DCs were washed twice with DPBS and resuspended in cold staining buffer (DPBS containing 2% heat-inactivated FBS) for 10 min blocking on ice. DCs were stained with APC-conjugated anti-HLA-DR [clone G46-6, mouse IGg2a, κ; BD Pharmingen, San Jose, CA], PE-conjugated anti-CD11c [clone B-ly6, mouse IGg21, κ; BD Pharmingen], and one of the following markers: a) CD1c [clone AD5-8E7, mouse IGg2a: Miltenyi Biotech]; b) CD14 [clone M5E2, mouse IGg2a, κ; BD Pharmingen]; c) CD80 [clone L307.4, mouse IGg1, κ; BD Pharmingen]; d) CD83 [clone HB15e, mouse IGg1, κ; BD Pharmingen]; e) CD86 [clone 2331 (FUN-1), mouse IGg1, κ; BD Pharmingen]; f) CD40 [clone 5C3, mouse IGg1, κ; BD Pharmingen]; g) MHC Class I [clone G46-2.6, mouse IGg1, κ; BD Pharmingen]; h) CCR7 [clone 3D12, rat IGg2a, κ; BD Pharmingen], or i) isotype controls (PE-, APC-, or FITC-conjugated mouse/rat IgG, κ; BD Pharmingen) for 30 min on ice. Cells were washed twice with staining buffer before analysis. For DC-LAMP expression, DCs were first stained with FITC-conjugated anti-HLA-DR and then subjected to fix-permeabilization treatment for 30 min at 4°C, and followed by intracellular staining with APC-conjugated anti-DC LAMP [clone I10-1112, mouse IGg1, κ; BD Pharmingen] for another 30 min on ice. Cells were washed twice with staining buffer and acquired on the same day on a BD Canto flow cytometer or BD Calibur (Becton Dickinson, Franklin Lakes, NJ). Data were analyzed using the Pro CellQuest software. DCs were first gated using the forward and side scatter dot plots, and the cell population highly expressing HLA-DR and CD11c was further analyzed for CD1c, CD14, CD80, CD83, CD86, CD40, CCR7, MHC Class I and DC-LAMP.

### IL-12p70 production by DC

DCs were cocultured with HOCl-oxidized SKOV3 lysate at a ratio of 1 DC to 1 tumor cell for 20 to 24 h at 37°C, 5% CO_2_. Fresh GM-CSF (500 IU/ml) was added to facilitate tumor lysate uptake by the DCs. Then 60 EU/ml of LPS and 2000 IU/ml of IFN-γ were added to the cocultures for 6 to 16 h to stimulate DC maturation. Supernatants of cocultures were collected by centrifugation and analyzed for IL-12p70 (i.e. IL-12p70 from fresh DCs). DCs from cocultures were also collected and washed twice with DPBS for further use. One half of these DCs were replated at 1 × 10^6 ^cells/ml in fresh complete CellGenix DC media supplemented with 2% human AB serum for a further 24 h to analyze for IL-12p70 (i.e. IL-12p70 from fresh replated DCs). The other half of the DCs were frozen in an infusible cryomedia or 90% human AB serum and then thawed for replating at 1 × 10^6 ^cells/ml in fresh complete CellGenix DC media supplemented with 2% human AB serum for 24 h to analyze for IL-12p70 (i.e. IL-12p70 from thawed replated DCs). IL-12p70 concentration was determined with ELISA according to the manufacturer's protocol (BioLegend, San Diego, CA). Briefly, NUNC MaxiSorp™ 96-well ELISA plates (Thermo Fisher Scientific, Waltham, MA) were coated with 1 × capture IL-12p70 for 2 h at 37°C, 5% CO_2 _or overnight at 4°C. Then, plates were washed 4 times with wash buffer (DPBS containing 0.05% Tween 20) using the ELISA plate washer (BioTek, Winooski, VT). Supernatants were diluted appropriately with 1x assay diluent (BioLegend) and incubated overnight at 4°C. The next day, the plates were washed 5 times with wash buffer and 1 × biotinylated detection antibody of IL-12p70 was added for 1 h incubation at room temperature. Plates were then washed 5 times with wash buffer and incubated with alkaline phosphatase-conjugated streptavidin for 30 min at room temperature. Five further washes with wash buffer were done and TBM substrate solution was added for 15 min. The reactions were stopped by adding equal volume of 1 N sulfuric acid (Fisher Scientific, Lawn, NJ). Plates were read using the ELISA plate reader (BioTek) and results expressed as pg/ml.

### IFN-γ intracellular staining

In some experiments, HER-2/neu or MART-1 specific T cells were used to assess the ability of DCs to present cognate antigen. HER-2/neu specific T cells were isolated from an HLA-A2^+ ^patient with breast cancer previously vaccinated with HLA-A2 restricted HER-2/neu peptides including HER-2/neu_369-377 _(KIFGSLAFL) [26]. HER-2/neu specific T cells were expanded by two rounds of incubation with MoDCs derived from HLA-A2^+ ^donors pulsed with HER-2/neu_369-377_. The frequency of HER-2/neu specific CD8 T cells in output T cells was 3-4%. MART-1 specific T cells were derived from tumor-reactive tumor-infiltrating lymphocytes after long-term culture in the presence of human recombinant IL-2, and were a kind gift of Dr. Stephen Rosenberg (Surgical Branch, National Cancer Institute, Bethesda, MD) [27].

DCs derived from HLA-A2^+ ^individuals were cocultured with HOCl-oxidized SKOV3 lysate at 1:1 cell ratio for 20 to 24 h at 37°C, 5% CO_2 _in the presence of fresh 500 IU/ml of GM-CSF. Then DCs were stimulated with 60 EU/ml of LPS and 2000 IU/ml of IFN-γ for 16 h. Two hours before the end of the LPS and IFN-γ stimulation (i.e. 14 h post-stimulation), DCs were pulsed with either HLA-A2 restricted HER-2/neu_369-377 _or MART-_126-35 _(EAAGIGILTV) [28] peptides (both from American Peptide Company, Inc., Sunnyvale, CA; ≥97% pure as determined by reverse-phase high performance liquid chromatography) at different concentrations (i.e. 0.1, 1 or 10 μg/ml) or with media (i.e. unpulsed for the last 2 h). Following that, DCs were harvested and cocultured with HER-2/neu or MART-1 specific T cell lines for a total of 5 h. After the 1^st ^hour of the total 5 h incubation, GolgiStop containing brefeldin A (1000x dilution according to manufacturer's instructions; BD Pharmingen) was added to the cocultures for the remainder of the incubation period. T cells were then harvested and stained for PE-Cy7-conjugated anti-CD8 (clone RPA-T8, mouse IgG1, κ; BD Pharmingen) followed by fix-permeabilization treatment (eBioscience, San Diego, CA) for 30 min at 4°C to stain intracellularly for IFN-γ (anti-IFN-γ PE-conjugated monoclonal antibody, clone 4S.B3, mouse IgG1, κ; BD Pharmingen). Then cells were washed twice with staining buffer (DPBS containing 2% heat-inactivated FBS), collected on the same day and interrogated using BD Canto flow cytometer (Becton Dickinson). Data were analyzed with Pro CellQuest software. T cells that were double-positive for CD8^+ ^and IFN-γ were expressed as a percentage of all the CD8^+ ^T cells.

### Cryopreservation and storage of mature lysate-loaded DCs

DCs that had been loaded with HOCl-oxidized SKOV3 lysate and subsequently matured with 60 EU/ml LPS and 2000 IU/ml IFN-γ for 6 to 16 h were cryopreserved at 5 × 10^6 ^DCs per ml of either a) infusible cryomedia that contained the following components in final concentrations: 31.25% Plasma-Lyte A, 31.25% D51/2NS (5% dextrose, 0.45% sodium chloride), 5% human serum albumin (HSA); 1% Dextran 40 (10% low molecular dextran in 5% dextrose), and 7.5% dimethyl sulfoxide (DMSO). Plasma-Lyte A injection (USP) and HSA were purchased from Baxter, Deerfield, IL. D51/2NS (USP) and low molecular dextran in 5% dextrose injection were obtained from Abbott Laboratories, North Chicago, IL. Cryoserve brand of DMSO was manufactured by Ben Venue Laboratories, Inc., Bedford, OH; or b) human AB serum (Valley Biomedical Inc.) containing 10% Hybri-Max™ sterile-filtered DMSO (Sigma-Aldrich Corp). The cells were frozen in a controlled-rate gradient freezer (model Forma 8018; Thermo Scientific, Asheville, NC).

### Cell viability

Cell viability was determined by Trypan Blue staining using a Bürker chamber (Paul Marienfeld GmbH & Co. KG, Lauda-Königshofen, Germany) and inverted phase microscope (Leica, Wetzlar, Germany). The % viable DCs was determined by expressing the number of live DCs as a percentage of the total number of DCs counted in the 4 big squares of the Bürker chamber.

### Mixed leukocyte reactions

DCs were cocultured with HOCl-oxidized SKOV3 lysate at a cell ratio of 1:1 for 20 to 24 h at 37°C, 5% CO_2 _in the presence of fresh 500 IU/ml of GM-CSF. Following that, 60 EU/ml of LPS and 2000 IU/ml of IFN-γ were added to the cocultures for 16 h to stimulate DC maturation. Then the cocultures were harvested, washed twice with DPBS and seeded in 96-well round bottom plates at 2.5 × 10^3^, 5 × 10^3 ^or 1 × 10^4 ^DCs/well. Allogeneic CD3^+ ^T cells from 3 different donors were added to the DCs at 1 × 10^5 ^cells/well. The cells were cultured for a total of 7 days at 37°C, 5% CO_2_. For the final 16 h of coculture, 1 μCi [^3^H] thymidine (ICN Biomedical, Costa Mesa, CA) was added. T cell proliferation was measured by liquid scintillation counting (Microbeta Systems; Immunotox, Richmond, VA). All assays were performed in triplicate. Control wells were set up to contain unpulsed immature DCs alone, unpulsed mature DCs alone or T cells alone. Results were expressed as counts per minute.

### Statistical analysis

Means for different experimental groups were analyzed from 3 to 6 independent experiments (i.e. DCs from 3 to 6 different individuals). The analysis of significance was carried out using unpaired Student's *t *tests or one-way ANOVA. A significance level of 0.05 or less was considered statistically significant.

## Results

### Optimization of culture media and cytokine concentrations

**Figure 1:**
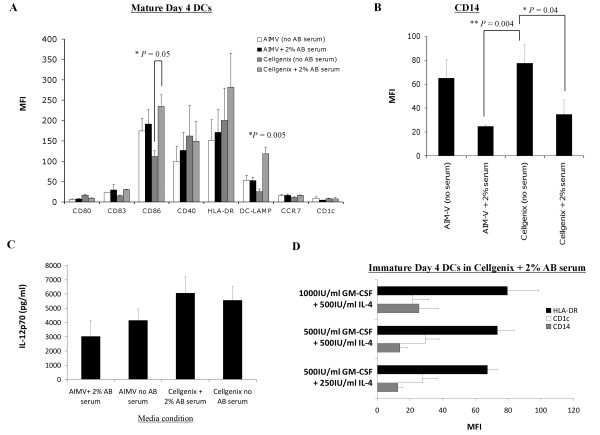
**Comparison of the phenotype and IL-12p70 production of mature HOCl-oxidized lysate-loaded DCs generated in human AB serum-free or serum-containing AIM-V and CellGenix DC media, and supplementation with different concentrations of GM-CSF and IL-4**. **(A-B) **DCs cultured in CellGenix DC media containing 2% human AB serum displayed a desirable phenotype with the highest level of DC-LAMP (*P = 0.005; one-way ANOVA) when compared to DCs cultured in other media conditions, and significantly higher CD86 (*P = 0.05) and lower CD14 (*P = 0.04) when compared to DCs cultured in serum-free CellGenix DC media. **(C) **DCs generated in CellGenix DC media plus 2% human AB serum produced slightly, though not significantly, higher amount of IL-12p70 compared to DCs generated in other media conditions (*P *= 0.2; one-way ANOVA). **(D) **500 IU/ml recombinant GM-CSF and 250 IU/ml recombinant IL-4 were sufficient concentrations for generating a favorable immature DC phenotype with low CD14, and intermediate HLA-DR and CD1c. All the data presented were the mean of 6 independent experiments (i.e. DCs from 6 different individuals) in research-scale cultures in Nunclon™Δ Surface T25 cm^2 ^flasks ± standard error of the mean (SEM). *P *values were determined with unpaired Student's *t *test unless otherwise stated. * denoted that *P *values were significant.

AIM-V and CellGenix DC media are two widely used clinical grade serum-free defined media for generating DCs [29-31]. Serum has been shown to improve DC differentiation and function [32] but the use of FBS could lead to transmission of bovine-related infectious agents, and the presence of serum contaminants could affect human DC differentiation and generate anti-bovine immunoreactivity that adversely impact administering multiple vaccinations [33-35]. Autologous serum is a possibility but the serum of cancer patients might contain high levels of inhibitory cytokines such as IL-6 [36] or IL-10 [37] that could affect DC function. Pooled human AB serum is a suitable alternative, where one batch of human AB serum can be used for an entire clinical trial to avoid batch-to-batch variation. Therefore, AIM-V and CellGenix DC media with or without 2% human AB serum for culturing DCs were compared. Research-scale DC preparations were set up by seeding each T25 cm^2 ^NUNC Surface flasks with 1 × 10^6 ^healthy donor monocytes/ml media (a total of 5 × 10^6 ^monocytes per flask). After 4 days of culture, DCs were loaded with HOCl-oxidized SKOV3 lysate for ~20 h, and matured with LPS and IFN-γ for ~16 h. DCs generated under the four different media conditions had overall comparable immunophenotypes, although DCs that were cultured with AB serum-supplemented CellGenix DC media exhibited the highest DC-LAMP (*P *= 0.04; one-way ANOVA) amongst all DCs, and expressed significantly higher CD86 (*P *= 0.05) when compared to DCs grown in serum-free CellGenix DC media [Figure 1A]. In addition, CD14 expression was significantly lower on DCs cultured in serum-supplemented media relative to matched serum-free media (AIM-V media, *P *= 0.004; CellGenix DC media, *P *= 0.04) [Figure 1B]. There were no significant differences in the amount of IL-12p70 protein secreted by DCs grown in the 4 different media conditions (*P *= 0.2; one-way ANOVA, Figure 1C).

Next, we analyzed the effect of different media on DC viability (Table 1). Viable DCs were counted (by Trypan blue exclusion) at the completion of four days of GM-CSF and IL-4 differentiation and then again at the end of pulsing with lysate and maturation with LPS and IFN-γ. CellGenix DC media containing 2% AB serum gave the highest final number of viable DCs compared to other media conditions (*P *= 0.016; one-way ANOVA, Table 1). These results collectively indicated that DCs cultured with CellGenix DC media containing 2% AB serum had a favorable immunophenotype of low CD14, high CD86 and DC-LAMP, high IL-12p70 capability, and high cell viability. Based on these results and the reported superior DC differentiation and function in the presence of serum [32], and the availability of CellGenix DC media in GMP grade, CellGenix DC media supplemented with 2% human AB serum was chosen as the leading media candidate for all subsequent experiments.

**Table 1:** DC viability following culture in four different media.

	**Number of viable DCs harvested from 5 × 10^6 ^monocytes per T25 cm2 flask**		
		
**Media condition**	**Before lysate-loading and maturation (10^6^)**	**After lysate-loading and maturation (10^6^)**	**% viable DC generated (± SEM)**
	
AIM-V no AB serum	4.52 ± 0.88	2.29 ± 0.36	53.39 ± 5.94
AIM-V + 2% AB serum	5.10 ± 0.92	3.05 ± 0.3	64.72 ± 7.14
CellGenix no AB serum	5.09 ± 0.61	2.76 ± 0.87	43.64 ± 6.17
CellGenix + 2% AB serum	5.48 ± 0.67	3.87 ± 0.35	73.28 ± 7.58*

CellGenix DC media supplemented with 2% human AB serum yielded the highest percentage of viable DCs after loading with HOCl-oxidized lysate (1 DC to 1 tumor cell ratio for 20-24 h) and 16 h maturation with LPS (60 EU/ml) and IFN-γ (2000 IU/ml). The final percentage of viable DC generated was calculated as follows: (number of viable DCs obtained per flask after lysate-loading and maturation ÷ number of viable DCs obtained per flask before lysate-loading and maturation) × 100%. The mean of 6 independent experiments ± standard error of the mean [SEM]) is shown. *P = 0.016; final % of viable DCs generated from CellGenix + 2% AB serum was significantly different from the final % of viable DCs generated with AIM-V no AB serum, AIM-V + 2% AB serum or CellGenix no AB serum (one-way ANOVA).

Next, we investigated the effect of different concentrations of recombinant GM-CSF (500 or 1000 IU/ml) and IL-4 (250 or 500 IU/ml) on DC differentiation and phenotype (Figure 1D). The levels of HLA-DR (black bar) and CD11c expression (white bar) were similar on immature DCs in all the conditions tested. No significant difference in CD14 (grey bar) expression was observed on DCs that were cultured in any of the conditions (*P *= 0.42, one-way ANOVA; Figure 1D). Thus, 500 IU/ml GM-CSF and 250 IU/ml IL-4 were selected for use in all subsequent DC preparations.

### Optimization of Culture Flasks

**Figure 2:**
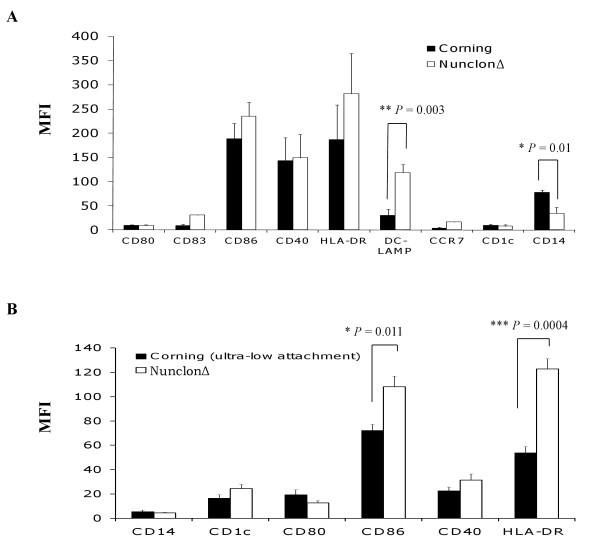
**Comparison of the effect of Nunclon™Δ Surface, Corning^® ^cell-culture surface and Corning^® ^ultra-low attachment surface on DC phenotype and differentiation**. DC were cultured with CellGenix DC media containing 2% human AB serum in T25 cm^2 ^flasks that had the same surface properties as one of the three commercially available cell factories - Nunclon™Δ Surface 1-tray Cell Factories, Corning^® ^CellSTACK^® ^culture chambers or Corning^® ^cell culture ultra-low attachment CellSTACK^® ^chamber. DC phenotype was evaluated following lysate-loading and maturation for 16 h with LPS and IFN-γ. **(A) **DCs cultured in Nunclon™Δ Surface flasks (surrogate of Nunclon™Δ Surface 1-tray Cell Factories) exhibited an overall favorable immunophenotype with significantly higher DC-LAMP (**P = 0.003) and lower CD14 (*P = 0.01) when compared to DCs cultured in Corning^® ^cell-culture surface flasks (surrogate of Corning^® ^CellSTACK^® ^culture chambers). Data were the mean of 6 independent experiments (i.e. DCs from 6 different individuals) in research-scale cultures ± SEM. **(B) **DCs that were cultured in Corning^® ^ultra-low attachment surface (surrogate of Corning^® ^ultra-low attachment cell factory chambers) expressed significantly lower CD86 (**P = 0.011) and HLA-DR (***P = 0.0004) following lysate-loading and maturation when compared to DCs cultured in Nunclon™Δ Surface (surrogate of Nunclon™Δ Surface 1-tray Cell Factories) and treated the same way. Data were the mean of 3 independent experiments (i.e. DCs from 3 different individuals) in research-scale cultures ± SEM. *P *values were determined with unpaired Student's *t *test. * denoted that *P *values were highly significant.

Cell factories are suitable for clinical-scale production of DCs for their large surface areas and as closed-cell culture systems to minimize product contamination. As each brand of cell factory might have different surface coating properties that could affect DC differentiation and maturation, three commercially available cell factories were evaluated - 1) Nunclon™Δ Surface 1-tray Cell Factories; 2) Corning^® ^CellSTACK^® ^culture chambers; and 3) Corning^® ^cell culture ultra-low attachment CellSTACK^® ^chamber. For ease of handling multiple DC preparations and assessing the reproducibility of the DC cultures, multiple research-scale DC preparations were set up in T25 cm^2 ^tissue culture flasks that had the same surface coating properties as the above cell factories. DCs were cultured in CellGenix DC media containing 2% human AB serum in all culture flasks, loaded with tumor lysate and matured with LPS and IFN-γ as described above. In Figure 2A, it was observed that DCs cultured in Nunclon™Δ Surface flasks (surrogate of Nunclon™Δ Surface 1-tray Cell Factories) exhibited an overall favorable immunophenotype with significantly higher DC-LAMP (*P *= 0.003) and lower CD14 (*P *= 0.01) when compared to DCs cultured in Corning^® ^flasks (surrogate of Corning^® ^CellSTACK^® ^culture chambers).

Next, Nunclon™Δ Surface was compared to Corning^® ^cell culture ultra-low attachment surface for generating DCs. Since Corning^® ^cell culture ultra-low attachment T25 cm^2 ^flasks (surrogate of Corning^® ^ultra-low attachment cell factory chambers) have hydrophilic and neutrally charged surface to inhibit cell attachment, it was hypothesized that this could facilitate easier DC harvesting compared to polystyrene Nunclon™Δ Surface. DCs were loaded with tumor lysate, and stimulated with LPS and IFN-γ as described above. Then, DCs were harvested with TrypLE™ Select and washed twice with DPBS before further analysis. DCs generated in Corning^® ^ultra-low attachment surface expressed significantly lower CD86 (*P *= 0.011) and HLA-DR (*P *= 0.0004) when compared to DCs generated in Nunclon™Δ Surface (Figure 2B). DCs generated in Corning^® ^cell culture ultra-low attachment surface were also smaller in size (data not shown) when compared to DCs cultured in Nunclon™Δ Surface flasks. These results suggested that DCs generated in Corning^® ^cell culture ultra-low attachment surface did not mature as efficiently in the presence of LPS and IFN-γ when compared to DCs cultured in Nunclon™Δ Surface. Nunclon™Δ Surface seemed to provide a suitable stratum for DC maturation and was chosen as the preferred vessel to generate DCs in subsequent experiments.

### Optimization of DC harvest

**Figure 3:**
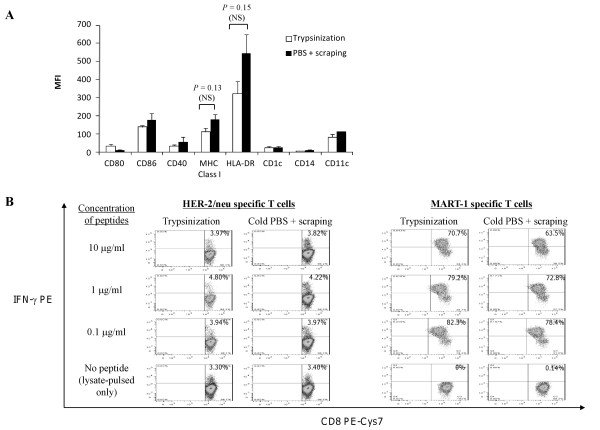
**Comparison of TrypLE™ Select animal-free tyrpsin immobilization and cold PBS with cell scraping for harvesting mature HOCl-oxidized lysate-loaded DCs**. **(A) **DCs were matured with LPS and IFN-γ for 16 h following lysate-loading. It was observed that mature lysate-loaded DCs that were harvested using TrypLE™ Select animal-free tyrpsin replacement exhibited approximately 30-40% reduction in the levels of MHC Class I (*P *= 0.13; NS) and HLA-DR (*P *= 0.15; NS) compared to DCs that were harvested with cold DPBS and cell scraping. Data were the mean of 3 independent experiments (i.e. DCs from 3 different individuals) ± SEM in research-scale Nunclon™Δ Surface T25 cm^2 ^flasks. *P *values were determined with unpaired Student's *t *test. NS indicated that *P *values were not significant. **(B) **Mature lysate-loaded DCs that were harvested with TrypLE™ Select animal-free tyrpsin replacement were as efficient as DCs that were harvested with cold DPBS and cell scraping in presenting MHC Class I-restricted HER-2/neu and MART-1 peptides and stimulating HER-2/neu and MART-1 CD8^+ ^T cells in intracellular IFN-γ assessments.

It was observed that approximately 50% of the Day-4 differentiated, immature and unpulsed DCs that were generated using Nunclon™Δ Surface (both T25 cm^2 ^flasks and 1-tray Cell Factories) in combination with CellGenix DC media supplemented with 2% human AB, would adhere firmly to the flask or cell factory surface (data not shown). After lysate loading and stimulation with LPS and IFN-γ, most DCs were firmly attached to the flask or chamber surface. Hence, a means to harvest these DCs was required. Two commonly used methods for harvesting mature lysate-pulsed DCs were evaluated: 1) by enzymatic mobilization using TrypLE™ Select, a recombinant fungal serine protease with trypsin-like activity derived from microbial fermentation (incubated for up to 10 min at 37°C); or 2) physical mobilization by incubation with cold DPBS for 30 min at 4°C followed by scraping. This experiment was conducted in Nunclon™Δ Surface T25 cm^2 ^flasks; DCs were loaded with oxidized whole tumor lysate followed by stimulation with LPS and IFN-γ as described above, and finally harvested for analysis. In Figure 3A, lysate-loaded, mature DCs that were harvested using TrypLE™ Select exhibited a modest (~30%) but not significant reduction in surface MHC Class I (*P *= 0.13) and HLA-DR expression (*P *= 0.15) compared to DCs that were harvested using cold DPBS and scraping. The levels of CD86, CD40 and CD11c on enzymatically mobilized DCs were similar to DCs mobilized using cold DPBS and scraping.

To determine whether enzymatic treatment affected the DC ability to present antigens to T cells, DCs were pulsed with HOCl-oxidized SKOV3 lysate and matured with LPS and IFN-γ. In the last 2 h of the 16 h DC maturation, lysate-loaded DCs were also pulsed with HLA-A2 restricted HER-2/neu_369-377 _or MART-1_26-35 _peptides at different concentrations (0.1, 1 or 10 μg/ml). DCs were harvested using TrypLE™ Select or by incubation in cold DPBS for 30 min at 4°C and followed by scraping. Harvested DCs were then cocultured for a total of 5 h with HLA-A2^+ ^T cells that comprised a measureable frequency of HER-2/neu specific T cells (derived from PBL of a breast cancer patient previously vaccinated with HER-2/neu_369-377 _[26]) or with MART-1 specific T cells [27]. DCs that had been harvested using TrypLE™ Select presented HER-2/neu (Figure 3B, left panel) and MART-1 (Figure 3B, right panel) peptides as efficiently as DCs that had been harvested with cold DPBS and scraping. Both DCs could present exogenous MART-1 peptides efficiently to CD8^+ ^T cells even at a low peptide concentration (i.e. at 0.1 μg/ml) and there was apparent saturation of MHC Class I sites at the lowest peptide concentration, as no further increase in IFN-γ T cell response could be detected with higher doses of either peptide [Figure 3B]. Importantly, DCs loaded with HOCl-oxidized SKOV3 ovarian tumor lysate but not pulsed with peptides efficiently presented endogenous HER-2/neu antigen to HER-2/neu specific CD8^+ ^T cells (but not to MART-1 specific CD8^+ ^T cells) [Figure 3B, left and right bottom panels], consistent with expression of HER-2/neu by SKOV3 cells. Neither T cells alone nor T cells cocultured with unpulsed DCs produced any IFN-γ (data not shown). These results show that DC enzymatic mobilization using TrypLE™ Select did not did not affect the ability of DCs to present class I restricted tumor-associated peptides.

Lastly, the viability of DCs mobilized with TrypLE™ Select or with cold DPBS and scraping was compared. Table 2 shows that the percentage of viable DCs harvested with TrypLE™ Select was significantly higher (*P *= 0.0003) compared to the more harsh method of harvesting DCs with cold DPBS and scraping. Thus, TrypLE™ Select is more suitable for mobilizing adherent DCs for clinical applications.

**Table 2:** DC viability following two different harvesting methods.

Harvesting method	% viable DC (± SEM)
Trypsinization	79.94 ± 3.66 ***
Cold PBS + scraping	59.47 ± 2.44

Trypsinization method yielded higher % of viable DCs compared to harvesting DCs with cold DPBS and scraping. Fresh monocytes were cultured in CellGenix DC media supplemented with 2% human AB serum for 4 days. Then immature DCs were loaded with oxidized tumor lysate for 20 to 24 h followed by maturation with LPS and IFN-γ for 16 h. The percentage of viable DC was determined by Trypan blue exclusion using the following formula: (total number of live non-Trypan blue DCs in all the four big squares of the Bürker chamber ÷ total number of live and dead DCs counted in the four big squares of the Bürker chamber) × 100%. Data is mean of 3 independent experiments (± SEM). *** *P *= 0.0003; highly significant difference was observed from cold DPBS + scraping (unpaired Student's *t *test).

### Optimization of DC cryopreservation

**Figure 4:**
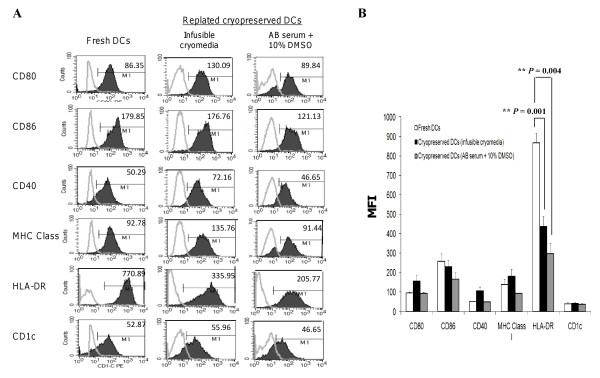
**Comparing the phenotype of DCs previously cryopreserved in infusible cryomedia or human AB serum containing 10% DMSO**. **(A) **Middle panel, DCs that had been cyropreserved with infusible cryomedia showed similar expressions of CD86 and CD1c as fresh DCs (first panel) and higher CD80, CD40 and MHC Class I levels after being thawed and replated for 24 h in fresh human AB serum-supplemented CellGenix DC media. Third panel, DCs that were cryopreserved with 90% human AB serum plus 10% DMSO exhibited similar level of CD80, CD40, MHC Class I and CD1c as fresh DCs. DCs cryopreserved in infusible cryomedia expressed approximately 50% lower levels of HLA-DR (**P = 0.001) compared to fresh DCs. On the other hand, DCs cryopreserved in human AB serum plus 10% DMSO media expressed approximately 80% lower levels of HLA-DR (**P = 0.004) compared to fresh DCs. Data were from a clinical-scale Nunclon™Δ Surface 1-tray Cell Factory and was representative of 3 independent research-scales experiments (i.e. DCs from 3 different individuals) in Nunclon™Δ Surface T25 cm^2 ^flasks. **(B) **Summary of flow cytometry results from the mean of 3 independent research-scales experiments in Nunclon™Δ Surface T25 cm^2 ^flasks (MFI ± SEM). *P *values are determined with unpaired Student's *t *test. ** denotes *P *value highly significant.

To overcome the need of preparing fresh lysate-pulsed DCs for each round of vaccination, freshly generated DCs could be cryopreserved after pulsing and maturation. DC stability was determined after cryopreservation in two types of cryomedia: 1) infusible cryomedia that contained Plasma-Lyte A, dextrose, sodium chloride injection, human serum albumin, and DMSO, and 2) 90% human AB serum plus 10% DMSO. Elutriated normal donor monocytes were cultured for 4 days in a Nunclon™Δ Surface 1-tray Cell Factory at 1 × 10^6^/ml to a total of 200 million cells per cell factory. Then differentiated DCs were loaded with HOCl-oxidized SKOV3 lysate at 1:1 cell ratio for 20-24 h plus 500 IU/ml GM-CSF, followed by 16 h stimulation with LPS and IFN-γ. After that, DCs were harvested using TrypLE™ Select and frozen in multiple aliquots at 5 × 10^6 ^DCs/ml cryopreservation media. Approximately 24 h later, the DCs were thawed and cultured in CellGenix DC media containing 2% human AB serum for 24 h and analyzed for viability (Table 3). Both types of cryomedia yielded similar high DC viability (92.9 ± 2.57% from infusible cryomedia and 86.5 ± 0.55% for AB serum with 10% DMSO, *P *= 0.6). The phenotype of the thawed DCs was analyzed by gating on 7-AAD negative viable cells (Figure 4A). DCs that had been cryopreserved with infusible cryomedia (Figure 4A middle column) retained similar levels of CD86 and CD1c as compared with fresh DCs (Figure 4A left column). HLA-DR surface expression on infusible cryomedia-cryopreserved DCs was significantly reduced as compared with fresh DCs (Figure 4B; *P *= 0.001). On the other hand, DCs that were cryopreserved with 90% human AB serum plus 10% DMSO retained similar levels of CD80, CD86, CD40, MHC class I and CD1c as that of fresh DCs (Figure 4A third column). These DCs had also significantly reduced levels of HLA-DR (Figure 4B; *P *= 0.004).

**Table 3:** DC viability following cryopreservation in two different cryomedia.

Cryopreservation method	% viable DC (± SEM)	
Infusible cryomedia	92.9 ± 2.57	P = 0.6 (NS)
90% human AB serum + 10% DMSO	86.5 ± 0.55	P = 0.6 (NS)

No significant difference in the percentage recovery of viable lysate-loaded DCs cryopreserved in infusible cryomedia or 90% human AB serum with 10% DMSO was observed post-thawed. Mature lysate-loaded DCs were frozen at 5 × 10^6 ^cells per ml in infusible cryomedia or 90% human AB serum plus 10% DMSO, and thawed 24 h later to determine the % of viable cell recovery. The % of viable DCs was calculated by Trypan blue exclusion using the following formula: (total number of live non-Trypan blue DCs in all the four big squares of the Bürker chamber total number of live and dead DCs counted in the four big squares of the Bürker chamber) × 100%. Data is the mean of 3 independent experiments (± SEM). *P *= 0.6 (NS; no significant difference in the viability of thawed DCs was observed when comparing the use of infusible cryomedia and 90% human AB serum with 10% DMSO) [unpaired Student's *t *test].

### Optimization of maturation length for DCs destined to cryopreservation

**Figure 5:**
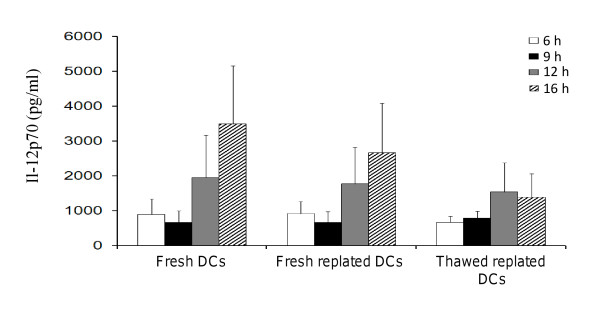
**Time-course of IL-12p70 production by DCs**. IL-12p70 was detected from fresh DCs from 6 h post LPS and IFN-γ stimulation and peaked at 16 h. Fresh replated DCs and thawed replated DCs continued to produce IL-12p70 albeit at a lower level than fresh DCs. Data were the mean of 3 independent experiments (i.e. DCs from 3 different individuals) in duplicates in research-scale Nunclon™Δ Surface T25 cm^2 ^flasks ± SEM. IL-12p70 concentration is expressed as pg/ml.

Robust production of IL-12p70 is a critical determinant of the potency of the DC vaccine *in vivo*. It has been shown to be essential for stimulating type I polarized T cells and inducing tumor-specific cytotoxic T cells by for tumor rejection [38-41]. Thus, the optimal time for mobilization of lysate-pulsed DCs after exposure to LPS and IFN-γ stimulation *in vitro *was determined in order to maximize the subsequent IL-12p70 production over the next 24 h (theorizing that this would be the critical period of T cell priming *in vivo *when DCs are injected into patients). Research-scale DC preparations were set up in Nunclon™Δ Surface T25 cm^2 ^flasks. After tumor lysate-loading, the DCs were treated with LPS and IFN-γ for 6, 9, 12 or 16 h and the cell culture supernatants collected for assessing IL-12p70 concentrations (thereafter called "fresh" DCs). These DCs were then harvested with TrypLE™ Select, thoroughly washed and immediately replated for 24 h in fresh media (thereafter called "fresh replated" DCs). Some DCs were frozen, then thawed 24 h later and replated for a further 24 h in fresh media (thereafter called "thawed replated" DCs). IL-12p70 was measured in the supernatants of fresh, fresh replated or thawed replated DCs. Fresh DCs started to produce IL-12p70 at 6 h (Figure 5) and continued to increase IL-12p70 production at 12 h (approximately 2000 pg/ml), and peaking at 16 h (approximately 3500 pg/ml). Fresh replated DCs were capable of producing IL-12p70 in the absence of further LPS and IFN-γ stimulation at levels comparable to fresh DCs (Figure 5). Encouragingly, thawed replated DCs were able to maintain their IL-12p70 production at similar levels as fresh DCs until 12 h, but some reduction was seen compared to 16 h fresh DCs. These results indicated that cryopreservation did not impair substantially IL-12p70 production from the DCs, and thawed DCs would be able to continue to produce IL-12p70 within the first 24 h after administration into cancer patients. In addition, the viability of the cryopreserved DCs were monitored after 24 h post-thawed to give an indication of the actual number of DCs that might survive *in vivo *in the patient after DC vaccine administered. It was found that DCs that had been treated with LPS and IFN-γ over a period of 6 to 16 h, cryopreserved in infusible media and thawed, showed comparable high viability of approximately 80% after 24 h of replating in fresh media (data not shown).

**Figure 6:**
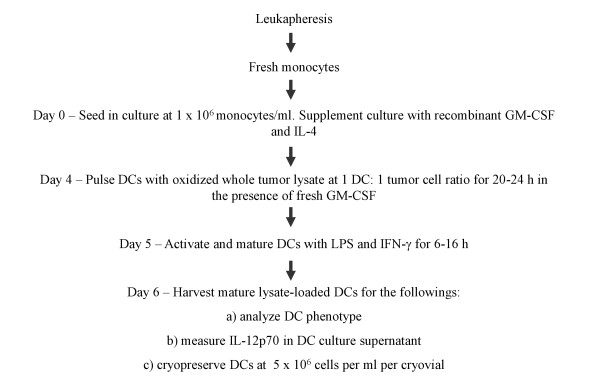
**Schematic diagram of optimized DC preparation**.

**Figure 7:**
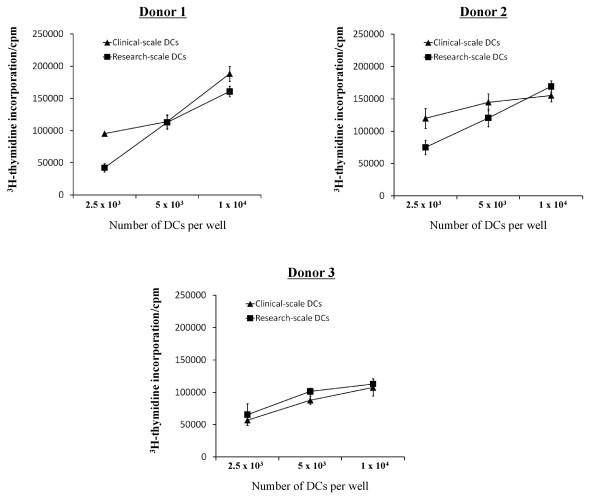
**Mixed leukocyte reaction capability of optimized DCs**. DCs generated from clinical-scale Nunclon™Δ Surface 1-tray Cell Factory and DCs generated from research-scale Nunclon™Δ Surface T25 cm^2 ^flasks using the optimized protocol depicted in Figure 6 were equally potent in stimulating robust mixed leukocytes reactions from 3 different individuals' T cells.

The results from this study provided an optimized methodology to produce tumor lysate-pulsed DCs that can be cultured to produce a mature phenotype, mobilized effectively, and cryopreserved with high viability while maintaining a robust mature DC phenotype and IL-12p70 production (summarized in Figure 6). We further tested the functionality of DCs prepared with the proposed methodology in mixed leukocyte reactions (MLRs) and compared the performance of DCs generated in research-scale T25 cm^2 ^culture flasks and in a clinical-scale cell factory system. DCs prepared and cryopreserved based on the final optimized protocol, were thawed and tested for their ability to stimulate the proliferation of allogeneic T cells from three different individuals. It was found that DCs, whether generated in a clinical-scale cell factory system or in research-scale T25 cm^2 ^culture flasks, were potent in stimulating robust T cell proliferation of allogeneic T cells in MLR (Figure 7).

## Discussion

Dendritic cell (DC)-based immunotherapy is a promising approach that has been used for over a decade in the clinic for treating malignancies. As DC generation procedures are highly variable and difficult to compare in many of these trials even within a given clinical setting or disease, there is a strong need to establish a defined set of robust criteria for generating DCs that are highly immunogenic and effective for the clinic. In this study we evaluated several important culture parameters for clinical-scale production that could highly influence the quality of the DC preparation, phenotype and function. In the process, choices were made based on significant results obtained under specific culture conditions or on obvious practical convenience when results were similar.

The first parameter investigated was the choice of media for preparing DCs. Many chemically-defined serum-free media are available including AIM-V media, CellGenix DC media and X-VIVO 10, 15 or 20 media. AIM-V [42-45] and CellGenix DC media [9,34,46] are selected for comparison because they are extensively used in DC clinical trials and are available in either therapeutic or GMP grade. It was observed that immature fully differentiated DCs generated in serum-free CellGenix DC media were non-adherent and smaller in size (similar to that of monocytes) compared to DCs cultured in serum-free AIM-V media (data not shown). However, DCs cultured in CellGenix DC media supplemented with 2% AB human serum showed larger size and exhibited the most favorable immunophenotype after lysate-loading and maturation with LPS and IFN-γ. These DCs expressed the highest levels of CD86 and DC-LAMP combined with low CD14 when compared to DCs cultured in other media conditions. These DCs also produced high amounts of IL-12p70 upon maturation. Although serum supplementation was not required for AIM-V media, as DCs generated in both serum-free and serum-supplemented AIM-V media were similar phenotypically, DCs generated in CellGenix DC media required serum supplementation to achieve optimal phenotype. The latter also showed significantly higher viability. Thus, CellGenix DC media supplemented with 2% human AB serum was chosen as the preferred media based on favorable DC size and immunophenotype, high IL-12p70 production and viability.

The second parameter investigated was the type of cell factory to use. Cell factories are especially suitable for clinical-scale production of DCs as they could be easily adapted in a GMP closed-culture system to avoid contamination, and be stacked to maximize space in the incubators. Each brand of cell factory may have different surface coating properties that could affect DC differentiation and maturation. Three commercially available cell factories were investigated- 1) Nunclon™Δ Surface 1-tray Cell Factories, 2) Corning^® ^CellSTACK^® ^culture chambers and 3) Corning^® ^cell culture ultra-low attachment CellSTACK^® ^chamber. It was observed that DCs cultured in Nunclon™Δ Surface exhibited a more favorable immunophenotype with significantly higher DC-LAMP and lower CD14 expression compared to DCs cultured with Corning^® ^cell-culture surface after lysate-loading and stimulation with LPS and IFN-γ. Interestingly, DCs generated using Corning^® ^ultra-low attachment surface did not mature properly with LPS and IFN-γ. These DCs showed lower CD86 and HLA-DR expression compared to DCs that were generated using the same type of media but with Nunclon™Δ Surface, thus highlighting the importance of DC attachment for proper differentiation and maturation in our cultures. This may also have important implications when considering the use of gas permeable culture bags where DCs bind less firmly to the bag surface compared to polystyrene-coated tissue culture surfaces. Additional work is required to optimize DC generation under less adhesive conditions especially with respect to IL-12p70 production [47]. In view of these findings, use of polystyrene Nunclon™Δ Surface is preferred.

At the completion of preparation, most of the mature lysate-loaded DCs were firmly attached to the flask or chamber surface. Hence a mean to harvest these DCs was required. The third parameter was to compare two commonly used methods for cell harvesting - by enzymatic mobilization or by incubation with cold DPBS for 30 min at 4°C followed by cell scraping. It was found that DCs were still strongly adhered to the culture flasks or cell factories after incubation with cold DPBS for 30 min at 4°C. Cold DBPS incubation alone was ineffective in detaching DCs and cell scraping was necessary. Cell scraping is a harsh physical method that can potentially damage important cell surface molecules and cause high rate of cell death. Indeed, only about 50% of DCs was viable after cell scraping. It is also not feasible to use in cell factories. The TrypLE™ Select is a recombinant trypsin that has similar kinetics and cleavage specificity as animal trypsin. It is produced in a controlled bacterial fermentation process that is completely free of animal- and human-derived components, thus minimizing the potential of contamination by mammalian pathogens. This property makes it highly suitable for use in DC preparations for the clinic. As TrypLE™ Select has the same biological activity as animal trypsin, i.e. to cleave adhesion molecules, there are concerns that important molecules such as HLA-DR and MHC class I required for T cell activation could be cleaved off from the DC surface. Although these molecules were detected at a somewhat lower level on DCs that had been treated with TrypLE™ Select compared to DCs treated with DPBS and cell scraping, these differences were not significant. However, enzymatically mobilized DCs had higher viability (82%) compared to DCs mobilized with scraping (50%). Importantly, enzymatically mobilized DCs were as capable as DCs that were detached with scraping to present HER-2/neu and MART-1 peptides at a low concentration (down to 0.1 μg/ml) to HER-2/neu and MART-1 specific T cells, respectively. Thus TrypLE™ Select was suitable for use to detach DCs without compromising the DCs viability or antigen presentation function.

DC therapy usually involves multiple rounds of vaccination and it can become very laborious to culture fresh DCs each time. Thus, DC cryopreservation is a highly desirable approach to minimize labor, time and costs of manufacturing. The feasibility of generating lysate-pulsed matured DCs in large numbers and cryopreserving them in -150°C until needed was assessed. Infusible cryomedia was compared to human AB serum plus 10% DMSO for freezing and storing lysate-pulsed mature DCs. It was found that the percentage of viable DCs obtained from cryopreservation with either infusible cryomedia or human AB serum was very similar (92.9% and 86.5%, respectively). These DCs also produced very similar levels of IL-12p70 after being thawed (not shown). However, infusible cryomedia was selected as the choice of freezing and storing DCs, since it contains less DMSO (7.5%) and it has been used in adoptive T cell therapy protocols, where thawed T cells were directly infused into patients without further washing [48]. Although the presence of small amount of DMSO is safe for patient administration and may not interfere with the function of T cells infused directly intravenously, in the case of DCs injected intradermally or intranodally, it is likely optimal to wash the thawed DCs twice with HBSS before administrating to patients.

In summary, several important clinical-scale parameters have been determined in this study - a) culturing DCs with GMP-grade CellGenix DC media supplemented with 2% human AB serum in NunclonΔ surface cell factory yielded DCs with the most favorable immunophenotype and high IL-12p70 production; b) 500 IU/ml GM-CSF and 250 IU/ml IL-4 was optimal for DC differentiation; c) TrypLE™ Select was suitable for harvesting DCs without impacting on DC viability and function; d) 6 to 9 h of DC stimulation with LPS and IFN-γ produced high level of IL-12p70, which continued after the cryopreserved DCs were thawed and replated for 24 h; and finally e) DCs cryopreserved with infusible cryomedia had high viability after being thawed and continued to produce high IL-12p70 for 24 h. Using the DC culture parameters determined above, a set of robust criteria based on DC phenotype (e.g. CD86, HLA-DR and CD14), IL-12p70 production *in vitro *and MLR for DC-vaccine immunogenicity could be proposed. Additional maturation/activation markers such as CD40, CD80, MHC Class I and CCR7 can be determined to give a more complete phenotypic profile of the DCs. Currently, this optimized method is being tested in a pilot phase clinical trial of autologous HOCl-oxidized tumor lysate-pulsed DC in patients with recurrent ovarian, primary peritoneal or fallopian tube cancer (NCT01132014) at the University of Pennsylvania Hospital.

## Conclusions

In this study, different culture parameters for clinical-scale DC preparation were optimized, including culture media, type of cell factory surface, method of harvesting DCs, type of cryomedia for freezing and storing DCs, and the duration of activating DCs with LPS and IFN-γ. The proposed final DC-vaccine product should express high levels of CD86 and HLA-DR, and low CD14. In addition, the DCs should be capable of producing IL-12p70 and be able to stimulate robust MLR *in vitro*. The optimized parameters determined in this study would further facilitate the development of a reference protocol for clinical-scale production of whole tumor lysate DC vaccines that is applicable to many forms of cancers.

## Competing interests

The authors declare that they have no competing interests.

## Authors' contributions

CLLC designed and performed all the experiments, analyzed the data and wrote the manuscript. DAM and ALB provided technical assistance to some research-scale and all clinical-scale DC experiments. DAM helped in analyzing some data. BLL, DJP, EL, QY and BJC contributed key reagents, materials and analysis tools. GC, LEK, BLL and DJP designed the experiments and supervised the study. GC conceived the study and finalized the manuscript. All authors have read and approved the final manuscript.
